# Bacteriostatic Effect of Some Plant Extracts Against Crown Gall Caused by *Agrobacterium tumefaciens* L.

**DOI:** 10.3390/ijms27020711

**Published:** 2026-01-10

**Authors:** Beata Jacek, Michał Miłek

**Affiliations:** 1 Department of Plant Physiology and Biotechnology, Faculty of Technology and Life Sciences, University of Rzeszów, Ćwiklińskiej 2, 35-601 Rzeszów, Poland; 2 Department of Chemistry and Food Toxicology, Institute of Food Technology and Nutrition, Faculty of Technology and Life Sciences, University of Rzeszów, Ćwiklińskiej 1a, 35-601 Rzeszow, Poland

**Keywords:** natural plant protection products, plant extracts, agar well diffusion method, bacterial disease, antimicrobial activity, flavonoids, polyphenols, HPTLC

## Abstract

The agar diffusion method was used to test the antibacterial activity of 12 plant species against *Agrobacterium tumefaciens*, the bacterium that is responsible for crown gall disease. Leaf, root, or flower extracts were prepared, but not all parts were used for each of the 12 plants listed. Plant extracts from leaves exhibited higher antibacterial activity than those from flowers and roots. Furthermore, the type of solvent had a significant influence on both the antibacterial activity and the flavonoid and polyphenol content. Acetone and alcohol extracts contained higher contents of these compounds than water extracts. The strongest bacteriostatic effect was of the leaf extracts of eucalyptus (*Eucalyptus nicholii* L.) and St. John’s wort (*Hypericum perforatum* L.). Based on HPTLC analysis, eucalyptus extracts contained, among others, chlorogenic acid, hyperoside, and quercetin, while St. John’s wort extracts contained rutin, hyperoside, and quercetin. The tansy leaf extracts (*Tanacetum vulgare* L.) were also rich in flavonoids and phenolic acids, such as kaempferol-3-glucoside, luteolin, chlorogenic acid, cynarine, and rutin. However, a moderate inhibitory effect against the tested bacterium was found in tansy extracts, as well as hop (*Humulus lupulus* L.), wormwood (*Artemisia absinthium* L.), peppermint (*Mentha piperita* L.), yarrow (*Achillea millefolium* L.), and nettle (*Urtica dioica* L.) extracts. The least effective were the root extracts of dandelion (*Taraxacum officinale* F.H. Wiggers coll.) and valerian (*Valeriana officinalis* L.), as well as the flower extracts of chamomile (*Matricaria chamomilla* L.) and marigold (*Calendula officinalis* L.). Given the lack of effective chemical products and the unavailability of commercially resistant cultivars, the use of plant-based extracts for protecting against crown gall appears to be of particular interest. The preliminary results are promising and suggest that eucalyptus and St. John’s wort extracts are the most promising for controlling *A. tumefaciens*.

## 1. Introduction

Bacterial plant diseases pose a significant threat to agricultural and horticultural production globally, resulting in substantial crop yield losses and economic damage [1]. Among the most well-known bacterial pathogens is *Agrobacterium tumefaciens*, which infects species in over 90 plant families worldwide [2,3]. It is a polyphagous bacterium with a wide host range, affecting woody and herbaceous plants, including forest and fruit trees, vegetables, and ornamentals [4].

*A. tumefaciens* is a Gram-negative, rod-shaped, motile, and soil-borne bacterium [3,5]. This bacterium causes crown gall disease, characterized by tumor-like gall formations on the roots of infected plants [5,6]. Plants with galls may be unable to transport water and nutrients properly. As a result, they become weakened and unproductive, and may eventually die [5,7]. Therefore, it is necessary to develop new, effective, and environmentally safe methods of crop protection against this disease.

Effective plant protection against bacterial diseases is a significant challenge due to the limited availability of chemical plant protection products. The management of crown gall disease is often restricted to preventive measures, such as using healthy propagation material, avoiding root injury, and maintaining good soil hygiene [5,8]. For this reason, biological methods can become increasingly important. A biological preparation containing the antagonistic bacterium *Agrobacterium radiobacter* K84 was introduced to the market [9]. However, this product is only effective against a limited number of *A. tumefaciens* strains and has a short-term effect [2,5]. Consequently, there is a need for new products in plant protection.

Plant extracts may represent a promising new approach in plant protection against pathogens. Plants are a rich source of bioactive compounds with potential bacteriostatic properties, including polyphenols, flavonoids, terpenoids, and lignans [10,11,12]. Different plant parts, such as leaves, stems, flowers, fruits, seeds, and roots, can be used for this purpose [13]. Many studies have reported that plant-derived natural compounds inhibit the growth of pathogenic organisms (e.g., [14,15,16,17,18,19]). The bacteriostatic properties of *Eucalyptus* species have been investigated in various studies, highlighting their potential as natural antimicrobial agents. Specifically, essential oil from eucalyptus plants was tested against human pathogens, including *Bacillus cereus*, *Escherichia coli*, *Staphylococcus aureus*, *Pseudomonas aeruginosa*, and *Enterococcus faecalis* [20,21,22,23,24,25]. In addition, the leaf extracts of *Eucalyptus camaldulensis* were tested against *Klebsiella* spp., *Salmonella typhi*, *Yersinia enterocolitica*, *Pseudomonas aeruginosa*, *Staphylococcus aureus*, and *Bacillus subtilis* [26]. However, information on the antibacterial activity of eucalyptus extracts against plant pathogenic bacteria are limited, with the exception of several studies [5,27,28]. Similarly, St. John’s wort has garnered attention for its antibacterial properties. Several studies have documented the bacteriostatic effects of St. John’s Wort against various pathogens, including *Escherichia coli*, *Staphylococcus aureus*, and *Salmonella enteritidis* [29,30]. Thus, St. John’s wort extracts may also be useful in reducing plant pathogens, but their potential is poorly understood and requires further investigation.

Many other plants have been studied for their bacteriostatic properties. For example, the antibacterial properties of common tansy have been the focus of recent research, highlighting its potential as a natural bacteriostatic agent. Tansy has been shown to be antibacterial against *Staphylococcus aureus* and *Pseudomonas aeruginosa* [31,32]. The yarrow also exhibits notable antibacterial activity against various bacterial species, including both Gram-positive and Gram-negative bacteria. Specifically, studies have found yarrow to be effective against pathogens such as *Staphylococcus aureus* and *Pseudomonas aeruginosa* [33]. These plants not only inhibit human pathogenic bacteria but potentially may inhibit plant pathogens.

Another plant with bacteriostatic properties may be nettle. Numerous studies have demonstrated that extracts from nettle plants possess substantial antibacterial activity against a range of pathogenic bacteria. For example, Salih et al. [34] reported significant antibacterial effects of aqueous and ethanol extracts of nettle against *Bacillus subtilis*, *Staphylococcus aureus*, and *Salmonella* spp. In addition, they indicated the presence of neophytadiene, which may play a crucial role in this antibacterial activity. In turn, Elez Garofulić et al. [35] highlighted that nettle leaves contain phenolic compounds which possess antimicrobial properties, enhancing their effectiveness against various bacterial strains.

Similarly, extracts of chamomile, dandelion, hop, peppermint, marigold, and wormwood have already been tested against *Staphylococcus aureus*, *Escherichia coli*, *Klebsiella pneumoniae*, or *Pseudomonas aeruginosa* [36,37,38,39,40,41]. Although the antimicrobial properties of plant extracts have been widely investigated in the context of human pathogens, there is still a lack of information on their potential effectiveness against plant pathogens. Therefore, the aim of the present study was to assess the bacteriostatic effectiveness of extracts from 12 plant species against *A. tumefaciens*.

## 2. Results

### 2.1. Bacteriostatic Properties

In the present study, extracts from 12 plant species were tested. Different plant parts were used for extraction, including leaves, roots, or flowers. The bacteriostatic activity of the tested extracts varied with plant part and solvent. Overall, leaf extracts showed higher antibacterial activity than flower or root extracts (Table 1). Moreover, four solvents—water, methanol, ethanol, and acetone—were used for extracting the active substances. The acetone and alcohol extracts were more effective than the water extracts. Especially the ‘MA’, ‘EA’, and ‘AWA2’ extracts showed the highest inhibitory activity against *A. tumefaciens*.

**Figure 1:**
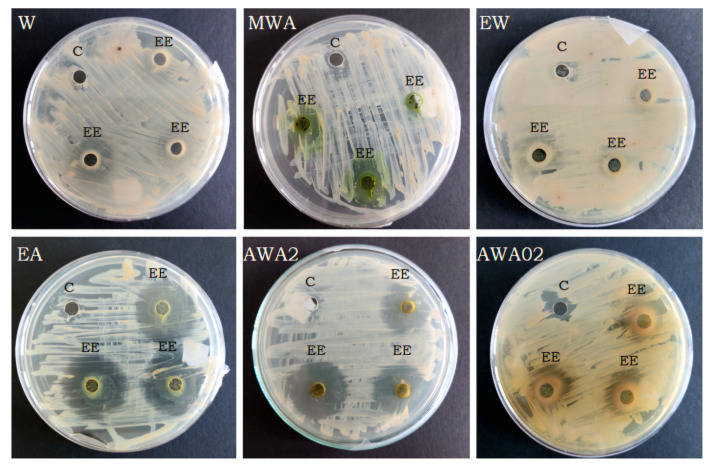
The antibacterial activity of eucalyptus plants, in relation to the solvent (C—control, EE—eucalyptus extracts).

In the present study, eucalyptus leaf extracts showed significantly the strongest inhibitory activity against *A. tumefaciens*, among the 12 plant species tested (Table 1). The average diameter of the growth inhibition zone was over 12 mm. However, the type of solvent significantly influenced antibacterial activity (Table 1, Figure 1). The ethanol extract with acetic acid (‘EA’) exhibited the highest zones of inhibition, measuring over 27 mm. High antimicrobial activity was also observed for the acetone extract ‘AWA2’, with a growth inhibition zone of almost 25 mm. The methanol extract with acetic acid (‘MA’) was also effective, with an inhibition zone exceeding 17 mm. In contrast, for extracts without acetic acid (‘W’, ‘MW’, and ‘EW’), the inhibition zone diameter did not exceed 1 mm.

Extracts of St. John’s wort leaves have also been tested against *A. tumefaciens*. The average diameter of the growth inhibition zone was almost 10 mm. Furthermore, the type of solvent significantly impacts their effectiveness (Table 1). Acetic acid-based acetone extracts had high bacteriostatic activity. Their growth inhibition zone diameter ranged from approximately 10 to 22 mm. Ethanol and methanol extracts containing acetic acid also showed a high growth inhibition zone, over 13 mm. However, water extracts were the least effective and showed no antibacterial activity against *A. tumefaciens*.

**Figure 2:**
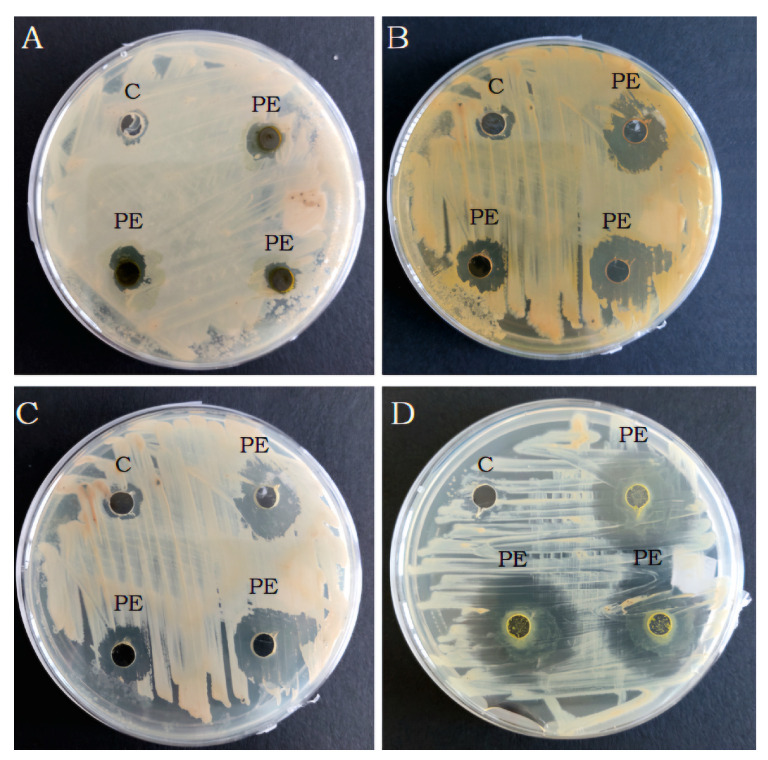
Comparison of antibacterial activity of different plant species (ethanol extracts of (**A**)—dandelion, (**B**)—tansy, (**C**)—yarrow, and (**D**)—eucalyptus, where C—control and PE—plant extracts).

Antibacterial potential of several other plant species against *A. tumefaciens* was also observed (Table 1, Figure 2). Leaf extracts of tansy, yarrow, wormwood, peppermint, and nettle, as well as the hop flower extracts, showed moderate inhibition. The average diameter of the growth inhibition zone ranged from almost 4 to 7 mm. Among these, tansy leaf extracts had the largest inhibition zone, followed by hop, yarrow, wormwood, and peppermint extracts (Table 1). The weaker activity was found for nettle leaf extracts. Nevertheless, significant differences were found among the tested solvents. In general, the ‘AWA2’ extracts had the highest bacteriostatic activity among other solvents. The ‘MA’ and ‘EA’ extracts also showed promising effects in inhibiting bacterial growth.

Four other plant species showed minimal antibacterial effect against the tested bacterium. These were root extracts of common dandelion and valerian, and flower extracts of common chamomile and marigold. The average diameter of the growth inhibition zone did not exceed 2 mm. In particular, *A. tumefaciens* was the least susceptible to valerian root extracts.

### 2.2. Total Flavonoid and Polyphenol Content

The total flavonoid content was different among plant species. The extracts of eucalyptus and St. John’s wort leaves are particularly rich in flavonoids (Table 2). The tansy extracts also contain higher content of these compounds than extracts of nine other plant species. However, the root extracts contained significantly lower flavonoid content than the flower and leaves extracts. The total flavonoid content of root extracts from common dandelion and valerian was much lower than that of leaf extracts from eucalyptus and St. John’s wort. The total flavonoid content in plant extracts also depended on the solvent type (Table 2). Higher flavonoid content was found in acetone and alcohol extracts, while the lowest was in water extracts.

The total polyphenol content in plant extracts was determined using the Folin–Ciocâlteu method. Significantly, the highest polyphenol content was found in eucalyptus leaves. High content of these compounds was also found in tansy and St. John’s wort extracts. The polyphenol content depended on the part of the plant from which they were isolated. The root extracts contain significantly less polyphenols than the flower and leaves extracts. The polyphenol content in plant extracts also depended on the solvent type. In general, acetone plant extracts contain more of these compounds than other extracts (Table 3). However, the lowest polyphenolic compound content was found in the water extracts.

### 2.3. HPTLC Analyses

**Figure 3:**
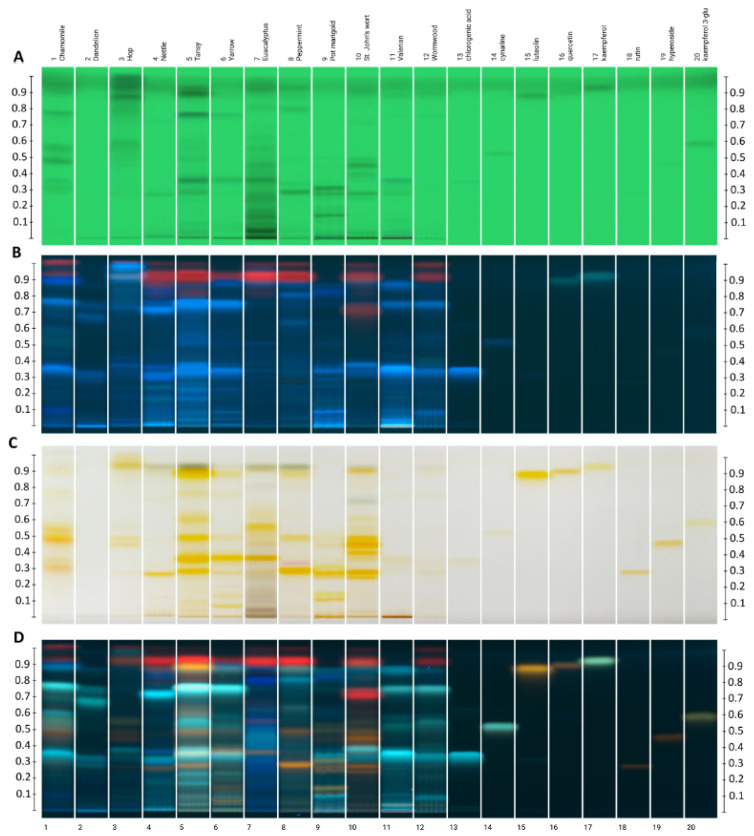
Image of HPTLC plate visualized: (**A**)—UV 254 nm before derivatization, (**B**)—UV 366 nm before derivatization, (**C**)—white light after derivatization, (**D**)—UV 366 nm after derivatization. Tracks 1–12: extracts, tracks 13–20: standards.

High-Performance Liquid Chromatography (HPLC) profiling of the plant acetone extracts revealed a rich spectrum of bioactive metabolites, predominantly phenolic acids and flavonoids (Table 4, Figure 3). The assignment of individual bands to phytochemical groups was made based on the color of the bands after derivatization with the Natural Product reagent/PEG 400 system. Generally, phenolic acids then take on a blue color, while flavonoids exhibit an orange-yellow color [42]. High amounts of active compounds were detected in tansy leaves. Among the studied extracts, tansy plants were the only ones that contained kaempferol-3-glucoside and luteolin. Three other compounds were also revealed, including chlorogenic acid, cynarine, and rutin. A high amount of flavonoids and phenolic acids was also separated in chamomile and St. John’s wort. Additionally, chlorogenic acid was confirmed in chamomile flower extracts, while rutin, hyperoside, and quercetin were identified in St. John’s wort leaf extracts. Eucalyptus leaf extracts were also found to be rich in flavonoids and phenolic acids, including chlorogenic acid, hyperoside, and quercetin.

A large quantity of phenolic compounds was extracted from yarrow leaves. Among the polyphenols, chlorogenic acid was identified. Phenolic acids were predominant in nettle leaf extracts, and rutin was identified. A high proportion of phenolic acids was also found in valerian root extracts. Chlorogenic acid and cynarine were the dominant substances identified. Similarly, phenolic acids, including chlorogenic acid, were found in wormwood leaf extracts.

In contrast, the flavonoids dominated in hop flower extracts, and rutin and hyperoside were revealed. In turn, dandelion root extracts contained lower content of phenolic acids and flavonoids than other plant extracts. Furthermore, leaf extracts exhibited high chlorophyll content. Most samples showed one red band on the chromatogram, except for the root extracts of dandelion and valerian and the flower extracts of marigold.

### 2.4. Cluster Analyses

**Figure 4:**
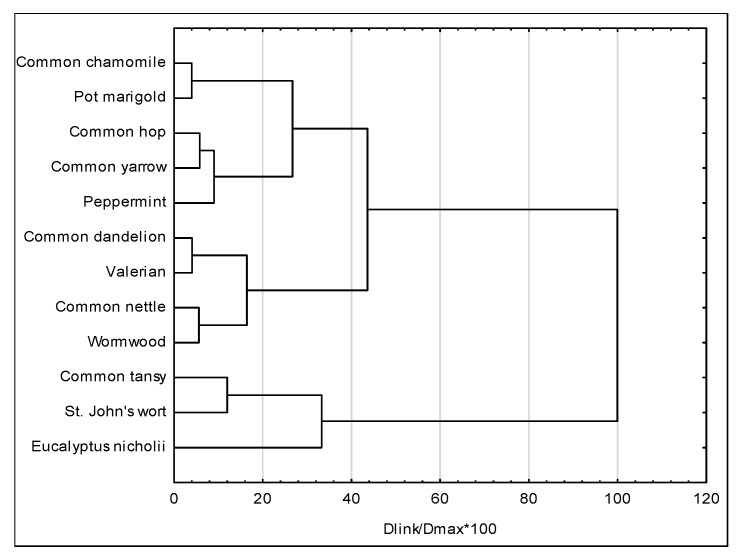
Dendrogram of the tested plant extracts’ similarity.

Cluster analysis divided the studied plant extracts into three groups (Figure 4). The first group consisted of extracts from eucalyptus, St. John’s wort, and tansy. These three extracts were clearly distinguishable from the others based on their growth inhibition zones and flavonoids and polyphenols content. The second group comprised wormwood and nettle extracts, as well as valerian and dandelion extracts. Peppermint, yarrow, and hop formed a third cluster. Additionally, this group included marigold and chamomile extracts, exhibiting the least bacteriostatic activity and the lowest total flavonoid and polyphenol content.

**Figure 5:**
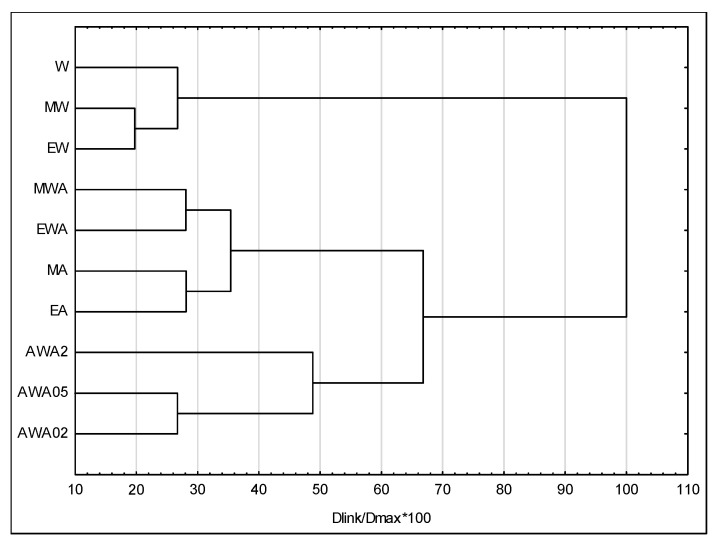
Dendrogram of extraction solvents’ similarity.

Ten extracts were divided into three groups based on cluster analysis (Figure 5). The first group consisted of the acetone extracts ‘AWA05’, ‘AWA02’, and ‘AWA2’. These acetone-acetic extracts exhibited the greatest inhibition zone of bacterial growth and a high content of flavonoids and polyphenols. The second group consisted of alcohol-acetic solvent extracts, including ‘EWA’, ‘MWA’, ‘EA’, and ‘MA’. However, the ‘W’, ‘MW’, and ‘EW’ extracts were separated from the other solvents.

## 3. Discussion

Plant-based active compounds can have antifungal and antibacterial properties [13]. However, there are no unified methods for preparing plant extracts, and factors such as extraction technique, solvent type, extraction time, sample weight, plant species, and plant parts all influence bacteriostatic activity.

Different solvents are selective for various plant compounds [43]. Thus, the potential effects of five solvents—water, methanol, ethanol, acetone, and acetic acid—were tested. The acetone and alcohol extracts were more effective than the water extracts. The ‘MA’, ‘EA’, and ‘AWA2’ extracts in particular showed the highest inhibitory activity against the tested bacterium. It can be supposed that the addition of acetic acid improves the bacteriostatic properties of plant extracts. Acetic acid is a weak organic acid whose antimicrobial action is mainly attributed to its ability to lower environmental pH. Moreover, it generates undissociated form that penetrates microbial cells, thereby leading to increased outer membrane permeability, intracellular acidification, and inhibition of pathogen growth [44]. Additionally, the specific part of the plant from which the active compounds are isolated is important. Leaves, roots, stems, bark, and flowers can contain different types and concentrations of bioactive compounds [45,46]. In the present study, extracts from flowers and roots showed lower antibacterial activity compared to leaf extracts.

Among the 12 plant species tested, eucalyptus leaf extracts showed the most potent inhibitory activity against *A. tumefaciens*. Previous studies have reported that eucalyptus essential oil was tested against a wide range of human pathogens, including *Bacillus cereus*, *Escherichia coli*, *Staphylococcus aureus*, *Pseudomonas aeruginosa*, and *Enterococcus faecalis* (e.g., [20,21,22,23,24,25]). Conversely, there are few reports on the antibacterial activity of eucalyptus extracts against plant pathogenic bacteria (e.g., [5,27,28]). With the exception of Kahla et al. [5], who demonstrated the effectiveness of ethyl acetate extract from *Eucalyptus cinerea* leaves in combating *A. tumefaciens*. In turn, Montesinos et al. [27] tested eucalyptus essential oil against *Xylella fastidiosa*, *Erwinia amylovora*, *Pseudomonas syringae*, and *Xanthomonas arboricola*. On the other hand, Pinto et al. [28] tested leaf water extracts of *Eucalyptus globulus* against *Pseudomonas syringae* pv. *tomato*, *Xanthomonas euvesicatoria*, and *Clavibacter michiganensis michiganensis*. This study provides additional information on the effectiveness of eucalyptus extracts against *A. tumefaciens*. The average diameter of the growth inhibition zone was 12 mm. However, the type of solvent significantly influenced the antibacterial activity. The ethanol extract with acetic acid (‘EA’) exhibited the highest zones of inhibition, measuring over 27 mm. High antimicrobial activity was also found in the acetone extract ‘AWA2’, with a growth inhibition zone measuring almost 25 mm. The methanol extract with acetic acid (‘MA’) was also effective, with an inhibition zone over 17 mm. These results are consistent with those of a previous study by Kahla et al. [5], who reported a bacteriostatic effect of eucalyptus leaf extracts against *A. tumefaciens* with inhibition zones ranging from 0 to 15 mm, depending on the solvent used. The results of the present study also confirm the bacteriostatic properties of eucalyptus plants. According to Kahla et al. [5], the high bacteriostatic activity of eucalyptus leaf extracts against *A. tumefaciens* may be due to the presence of terpenoids like cineole, camphene, pinene, and terpineol. Eucalyptus leaves are also rich in flavonoids and phenolic compounds [47].

St. John’s wort is a valuable medicinal plant due to its secondary metabolites, including flavonoids, tannins, and hypericin [48]. Several studies have reported the bacteriostatic effects of St. John’s wort against various pathogens, including *Escherichia coli*, *Staphylococcus aureus*, *Enterococcus faecalis*, and *Salmonella enteritidis* [32,33]. These results suggest that St. John’s wort extracts can inhibit the growth of phytopathogenic bacteria. Thus, the effect on *A. tumefaciens* was tested in the present study. The average diameter of the growth inhibition zone was almost 10 mm. Furthermore, the effectiveness was significantly influenced by the type of solvent. Acetic acid-based acetone extracts (‘AWA2’) had the highest bacteriostatic activity. The growth inhibition zone diameter was almost 22 mm. Ethanol and methanol extracts containing acetic acid also showed a large growth inhibition zone, approximately 13 mm in diameter.

Many other plants are widely known for their bacteriostatic properties. However, extracts of tansy, yarrow, hop, wormwood, peppermint, and nettle were moderately effective at inhibiting the growth of *A. tumefaciens*. Tansy is a perennial herbaceous plant known for its antibacterial and antifungal properties. It contains active compounds, which are a promising starting point for developing new antimicrobials [49]. Recent investigations have demonstrated the antibacterial efficacy of tansy against *Staphylococcus aureus*, *Escherichia coli*, and *Pseudomonas aeruginosa* [31,32]. This study also found bacteriostatic activity of tansy against *A. tumefaciens*. The average diameter of the growth inhibition zone was almost 7 mm. Conversely, acetone extracts ‘AWA2’ and ‘AWA05’ exhibited significant antibacterial activity, with inhibition zones of 17 mm and 13 mm, respectively. This suggests that acetone is more effective at extracting active compounds from the studied plants, thereby enhancing their antimicrobial potential. However, the third acetone solvent (‘AWA02’) showed no bacteriostatic activity. Alcohol-acetic solvents were also effective, as demonstrated by the 11 mm inhibition zone of the methanol extract ‘MA’.

The yarrow plants exhibited moderate antibacterial activity against *A. tumefaciens*. The average diameter of the growth inhibition zone exceeded 6 mm. However, after using the acetone solvents ‘AWA2’, the zone of bacterial growth inhibition exceeded 14 mm. Yarrow contains many active compounds, which may contribute to its antimicrobial properties [50]. However, no information was found in the available literature about the bacteriostatic activity of yarrow plants against *Agrobacterium tumefaciens*. On the other hand, the hexane, chloroform, acetone, and methanol extracts of common yarrow showed weak antibacterial activity against 25 phytopathogenic bacteria, including *Erwinia amylovora*, *Erwinia carotovora*, *Pseudomonas syringae*, and *Xanthomonas campestris* [51]. Hop flower extracts also moderately inhibited the growth of *A. tumefaciens*. According to Kornyšova et al. [52], hop contain humulone and lupulone. These compounds exhibit potent antimicrobial activity against most of the tested microorganisms, indicating their potential for the development of plant-based antimicrobial agents [53].

By contrast, extracts of wormwood and peppermint exhibited weak antibacterial activity against *A. tumefaciens*. Similarly, nettle extracts also showed an even weaker inhibitory effect. Nevertheless, extracts from four plant species showed minimal antibacterial effect against *A. tumefaciens*. These were root extracts of common dandelion and valerian, and flower extracts of common chamomile and marigold. The average diameter of the growth inhibition zone did not exceed 2 mm. Notably, *A. tumefaciens* exhibited the least susceptibility to valerian root extracts.

Flavonoids and polyphenols are key secondary metabolites with antibacterial properties. Tagousop et al. [54] and Liga et al. [55] showed that flavonoids can disrupt cell membranes, inhibit nucleic acid synthesis, and paralyze bacterial energy metabolism. However, flavonoid content varies by plant species. Eucalyptus, St. John’s wort, and tansy extracts had high concentrations of these compounds. In general, extracts from flowers and leaves had more flavonoids than extracts from roots. The extraction solvent also influenced total flavonoid content, with acetone being the most effective. Munhoz et al. [56] and Sasadara and Wirawan [57] also reported higher total flavonoid content in acetone extracts than in water extracts, which is consistent with our results. Water extracts are less effective at extracting many antimicrobial compounds than organic solvents and may have a weak inhibitory effect on bacteria.

Polyphenols also exhibited antibacterial properties, affecting a wide range of bacteria and fungi. These compounds damage the cytoplasmic membrane, cell wall, nucleic acids, and enzymes of microorganisms [58]. Similarly, the polyphenol content in the plant varies depended on the species. Extracts from eucalyptus and St. John’s wort are richer in polyphenols than those from other plants. Acetone was the most effective solvent for extracting polyphenols, resulting in higher bacteriostatic properties. Similarly, Michiels et al. [59], Abozed et al. [60], Złotek et al. [61], and Dirar et al. [62] also recommended the use of acetone solvent for polyphenol extraction. On the other hand, Tomsone et al. [63], Salih et al. [64], and Palaiogiannis et al. [65] reported that alcohol extract was more effective in extracting polyphenolic compounds.

Eucalyptus leaves are rich in flavonoids and phenolic compounds [47], as confirmed by HPTLC analysis. Leaf extracts of *Eucalyptus nicholii* were found to contain chlorogenic acid, hyperoside, and quercetin. Previously, Moges et al. [47] in *Eucalyptus* sp. identified the phenolic compounds such as quercetin, kaempferol, luteolin, phloretin, catechins, rutin, ellagic acid, hydroquinone, protocatechuic acid, naringenin, chlorogenic acid, hesperetin, pyrogallol, and resorcinol. According to Kahla et al. [5], eucalyptus plants contain additional compounds other than polyphenols and flavonoids with bacteriostatic effects, including cineole, pinene, terpineol, and limonene.

According to Kazlauskas and Bagdonaite [66], the main active substances of St. John’s wort are rutin, quercetin, and hypericin. These compounds were also identified in the present study. Furthermore, the authors additionally identified isoquercetin. Compounds such as polyphenols, flavonoids, and terpenoids are responsible for the bacteriostatic properties and antioxidant effects of many plant extracts. Thus, the presence of these compounds in eucalyptus and St. John’s wort largely explains their potent bacteriostatic activity against *A. tumefaciens*.

Tansy is also rich in phenolic acids and flavonoids, which exhibit bacteriostatic properties. This study identified the following biologically active substances in tansy leaves: kaempferol-3-glucoside, luteolin, chlorogenic acid, cynarin, and rutin. Significant amounts of luteolin, chlorogenic acid, and rosmarinic acid were also reported in tansy leaf by Babich et al. [67]. In turn, Keskitalo et al. [49] analyzed the compounds present in tansy and identified camphor, thujone, cineole, and davadone-D.

A large number of phenolic compounds were also isolated from yarrow leaf extracts in the present study. The main component was chlorogenic acid. Székely-Szentmiklósi et al. [50] also identified dicaffeoylquinic acids and flavonoids in yarrow plants. In turn, rutin and hyperoside were revealed in hop flower extracts. These compounds were also confirmed by the study of Latypova et al. [68]. However, other flavonoids were also found in hop leaf extracts, including cynaroside, dihydroquercetin, quercetin, kaempferol, and luteolin. These results suggest that hop leaves may have a higher bacteriostatic potential than flowers, and could serve as an additional source of polyphenolic compounds. In turn, the study of Kornyšova et al. [52] focused on the hop bitter acids, such as humulone and lupulone.

Notably, HPTLC analysis confirmed that the lowest levels of polyphenols and flavonoids were found in dandelion and valerian roots. This explains their limited bacteriostatic effect on *A. tumefaciens* growth. The flavonoids and phenolic acids found in chamomile, such as apigenin, luteolin, chlorogenic acid, caffeic acid, quercetin, and rutin, have been thoroughly documented [69]. These compounds have bacteriostatic properties. However, chamomile root extracts were also ineffective against *A. tumefaciens*.

This research was conducted under in vitro conditions. It is necessary to expand the study to include greenhouse tests and further field experiments. Nevertheless, the initial laboratory results are promising and suggest that eucalyptus and St. John’s wort extracts could be a valuable, natural alternative for controlling *A. tumefaciens*. However, further investigation into the extraction of biologically active compounds from plants and an evaluation of their phytotoxic effect is required. Furthermore, optimizing extraction methods and assessing the stability of the preparation will be essential to ensure its practical usefulness.

## 4. Materials and Methods

### 4.1. Plant Material and Sample Preparation

The plant material was purchased from the ‘Ziołowy Dwór’ herbal shop in Poland. According to the manufacturer’s information, the eucalyptus plants were collected in Albania, while the other plants were collected in Poland. Leaves, roots, or flowers of 12 plant species were dried at room temperature without sunlight, then ground in a laboratory mill (MMK-06M, MPM, Milanówek, Poland). Not all parts were used for each of the 12 plants listed (Table 5). The moisture content of the dried plant material was approximately 15–20%. The powdered plant material was extracted with five solvents: water, methanol, ethanol, acetone, and acetic acid (ACS reagent grade, Chempur) (Table 6). Two grams of plant material were dissolved in 30 mL of the solvent and shaken at 160 rpm in a shaking water bath (GFL 1092, Bionovo, Legnica, Poland) at room temperature for 24 h. The mixture was then filtered through Whatman No.1 filter paper (Sigma-Aldrich, St. Louis, MO, USA), and the extracts were stored at 4 °C until analysis.

*A. tumefaciens* C58 was obtained from the Phytopathology Workshop at the Institute of Horticulture in Skierniewice, Poland. The bacteriostatic properties of each plant extract were tested by the agar well diffusion method. Bacterial cultures were incubated in YEB medium (Thermo Fisher Scientific, Waltham, MA, USA) at 28 °C overnight and then adjusted to a 0.5 McFarland standard. Then, 100 µL of this suspension was evenly spread on Petri dishes. Four 5 mm diameter wells were made in the medium using a sterile cork borer. 100 µL of solvent without plant extract was added to the first well (control), and 100 µL of the tested plant extracts was added to the other three wells. The Petri dishes were incubated at 28 °C for 48 h in a laboratory incubator (Q-Cell, Pol-Lab, Wilkowice, Poland). The experiment included five replicates for each plant extract and solvent type. After this time, the diameter of the growth inhibition zone around each well was measured. The real growth inhibition zone of *A. tumefaciens* was obtained by subtracting the diameter of the zone of inhibition observed with the plant extracts from the diameter of the zone of inhibition caused by the solvent alone.

### 4.2. Total Flavonoid and Polyphenol Determination

The aluminum chloride method described by Yadav et al. [70] was used to determine total flavonoid content. The reaction mixture consisted of 0.1 mL of plant extract, 1.6 mL of methanol, 0.1 mL of 10% AlCl_3_·6H_2_O (ACS reagent grade, Chempur, Piekary Śląskie, Poland), 0.1 mL of 1M CH_3_COONa (ACS reagent grade, Chempur), and 2.8 mL of distilled water. After 40 min of incubation at room temperature, the absorption of the samples was measured at 415 nm on a Aquamate Plus UV/VIS spectrophotometer (Thermo Scientific, Waltham, MA, USA). The results were expressed as milligrams of quercetin equivalents per gram of plant dry mass. Total polyphenol content was determined using the Folin–Ciocâlteu method by Żbik et al. [71]. The reaction mixture consisted of 0.1 mL of plant extract, 6 mL of distilled water and 0.5 mL of Folin–Ciocâlteu reagent (Chempur). After three minutes, 1.5 mL of saturated sodium carbonate solution (ACS reagent grade, Chempur) and 1.9 mL of distilled water were added. After 30 min of incubation at 40 °C, the absorbance was measured at 765 nm using a Aquamate Plus UV/VIS spectrophotometer. These results were expressed as milligrams of gallic acid equivalents per gram of plant dry mass. Three replicates were performed for each sample to determine total flavonoid and polyphenol content.

### 4.3. HPTLC Qualitative Phytochemical Profiling

High-performance thin-layer chromatography (HPTLC) was used to qualitatively compare the phytochemical profiles of the studied extracts. Only extracts with the highest total phenol and flavonoid content were selected for comparison. Analysis was performed using an HPTLC kit from Camag (Muttenz, Switzerland) consisting of an automatic applicator (Linomat 5), an automatic developing chamber (ADC-2), a visualizer, and a derivatizer. The modified method previously described by Dżugan et al. [72] for the analysis of *Solidago* spp. extracts was used. HPTLC Silica Gel 60 F254 plates, 20 cm × 10 cm (Merck, Darmstadt, Germany) were used. A mixture of ethyl acetate, water, acetic acid, formic acid (15:2:1:1, *v*/*v*/*v*/*v*) was used as the developing phase. The samples were applied as 8 mm strips in a volume of 3 µL. Standards (chlorogenic acid, cynarine, luteolin, quercetin, kaempferol, rutin, hyperoside, kaempferol-3-glucoside; Sigma-Aldrich, St. Louis, MO, USA) were used at a concentration of 200 µg·mL^−1^ in methanol, loading volume was 2 µL. After development, the plate was heated for 3 min at 100 °C and then sprayed sequentially with Natural Product reagent solution and PEG 400 solution. The plate was visualized before derivatization under UV light (254 and 366 nm) and after derivatization (UV 366 and white light). Image analysis was performed using Vision CATS 3.2 software (CAMAG, Muttenz, Switzerland).

### 4.4. Statistical Analyses

Statistical analysis was performed using the Statistica program version 13.3. Analysis of variance ANOVA with the LSD mean separation test at a significance level of 0.05 was performed. The experimental results were presented as means ± standard deviation. Cluster analysis using Ward’s method and the Euclidean distance were also performed. The diameter of the growth inhibition zone, as well as total flavonoid and polyphenol content, was used for agglomeration analyses.

## 5. Conclusions

Given the lack of effective chemical products and the unavailability of commercially resistant cultivars, protecting plants against crown gall seems of particular interest. The studied plant extracts exhibited differential activity against *A. tumefaciens*. Eucalyptus and St. John’s wort extracts were the most effective at controlling *A. tumefaciens*, possibly due to their higher flavonoid and polyphenol content. Eucalyptus extracts contained chlorogenic acid, hyperoside, and quercetin, while St. John’s wort extracts contained rutin, hyperoside, and quercetin.

Moderate effectiveness was demonstrated for extracts of yarrow, tansy, hop, wormwood, peppermint, and nettle. In contrast, the least effective extracts were those from dandelion, valerian, chamomile, and marigold. It is worth emphasizing that the extracts from the leaves exhibited higher antibacterial activity than those from the flowers and roots. The bacteriostatic properties, as well as the flavonoid and polyphenol content, were significantly impacted by the solvent type used. Acetone and alcohol extracts contained higher contents of these compounds than water extracts, resulting in better bacteriostatic properties. Water extracts were less effective at extracting antimicrobial compounds than organic solvents and had a weak inhibitory effect on bacteria.

## Figures and Tables

**Table 1:** The real diameter of the growth inhibition zone of the tested plant extracts.

Plant	Plant Part	Solvent										$\bar{x}$
		W	MW	MWA	MA	EW	EWA	EA	AWA2	AWA05	AWA02	
	mm											
Common chamomile	F	0.0 ± 0.0 ^a^	1.0 ± 0.1 ^ab^	1.3 ± 1.0 ^bc^	3.3 ± 0.3 ^d^	1.4 ± 0.4 ^bc^	0.3 ± 0.3 ^ab^	2.3 ± 0.4 ^cd^	3.3 ± 0.6 ^d^	0.0 ± 0.0 ^a^	0.0 ± 0.0 ^a^	1.3 ± 1.5 ^A^
Common dandelion	R	0.0 ± 0.0 ^a^	1.0 ± 0.1 ^ab^	0.2 ± 0.1 ^ab^	1.7 ± 0.5 ^b^	0.1 ± 0.1 ^a^	1.2 ± 0.4 ^ab^	4.6 ± 1.0 ^c^	6.4 ± 1.1 ^d^	0.7 ± 0.4 ^ab^	0.7 ± 0.4 ^ab^	1.7 ± 2.3 ^AB^
Common hop	F	0.0 ± 0.0 ^a^	0.2 ± 0.1 ^a^	2.8 ± 0.2 ^b^	9.9 ± 1.3 ^e^	3.7 ± 0.3 ^b^	5.8 ± 0.3 ^c^	8.6 ± 1.1 ^d^	12.3 ± 0.8 ^f^	7.7 ± 0.2 ^d^	11.8 ± 1.0 ^f^	6.3 ± 4.3 ^DE^
Common nettle	L	0.0 ± 0.0 ^a^	0.2 ± 0.1 ^a^	2.1 ± 0.1 ^b^	8.6 ± 1.2 ^d^	0.0 ± 0.0 ^a^	1.6 ± 0.3 ^b^	6.6 ± 1.0 ^c^	13.3 ± 0.9 ^e^	1.7 ± 0.4 ^b^	1.5 ± 0.3 ^b^	3.6 ± 4.3 ^BC^
Common tansy	L	0.0 ± 0.0 ^a^	0.0 ± 0.0 ^a^	7.8 ± 0.5 ^b^	11.0 ± 0.5 ^c^	1.1 ± 0.3 ^a^	8.4 ± 0.4 ^b^	7.4 ± 0.3 ^b^	16.6 ± 2.1 ^e^	13.4 ± 1.3 ^d^	0.5 ± 0.1 ^a^	6.6 ± 5.8 ^E^
Common yarrow	L	0.0 ± 0.0 ^a^	0.0 ± 0.0 ^a^	7.8 ± 0.3 ^c^	10.6 ± 0.6 ^d^	0.0 ± 0.0 ^a^	6.0 ± 0.4 ^b^	9.9 ± 1.1 ^d^	14.8 ± 2.3 ^e^	10.5 ± 0.5 ^d^	0.5 ± 0.2 ^a^	6.0 ± 5.4 ^DE^
Eucalyptus nicholii	L	0.9 ± 0.4 ^a^	0.0 ± 0.0 ^a^	16.0 ± 1.6 ^d^	17.5 ± 1.6 ^d^	0.7 ± 0.3 ^a^	16.5 ± 0.8 ^d^	27.3 ± 1.2 ^f^	24.7 ± 1.6 ^e^	13.7 ± 2.7 ^c^	6.4 ± 0.3 ^b^	12.4 ± 9.6 ^G^
Peppermint	L	0.0 ± 0.0 ^a^	0.0 ± 0.0 ^a^	4.1 ± 0.6 ^b^	9.1 ± 1.6 ^c^	0.0 ± 0.0 ^a^	10.0 ± 1.8 ^c^	3.6 ± 0.4 ^b^	12.3 ± 2.1 ^d^	3.5 ± 0.4 ^b^	1.0 ± 0.5 ^a^	4.4 ± 4.5 ^CD^
Pot marigold	F	0.0 ± 0.0 ^a^	0.0 ± 0.0 ^a^	0.0 ± 0.0 ^a^	4.3 ± 0.3 ^d^	0.0 ± 0.0 ^a^	0.8 ± 0.2 ^bc^	0.4 ± 0.1 ^ab^	4.4 ± 0.3 ^d^	1.1 ± 0.3 ^c^	0.0 ± 0.0 ^a^	1.1 ± 1.7 ^A^
St. John’s wort	L	0.0 ± 0.0 ^a^	0.0 ± 0.0 ^a^	13.5 ± 1.3 ^cd^	13.1 ± 0.7 ^c^	0.1 ± 0.1 ^a^	12.9 ± 0.3 ^c^	15.4 ± 0.5 ^d^	21.4 ± 4.4 ^e^	11.8 ± 0.8 ^c^	9.6 ± 2.3 ^b^	9.8 ± 7.2 ^F^
Valerian	R	0.0 ± 0.0 ^a^	0.2 ± 0.1 ^a^	0.3 ± 0.1 ^a^	0.3 ± 0.3 ^a^	1.4 ± 0.4 ^b^	2.1 ± 0.1 ^b^	1.7 ± 0.3 ^b^	2.3 ± 0.4 ^b^	1.4 ± 0.8 ^b^	0.0 ± 0.0 ^a^	1.0 ± 1.1 ^A^
Wormwood	L	0.0 ± 0.0 ^a^	0.0 ± 0.0 ^a^	6.4 ± 1.2 ^c^	8.4 ± 1.7 ^d^	0.0 ± 0.0 ^a^	3.2 ± 0.7 ^b^	17.1 ± 2.3 ^f^	11.4 ± 1.7 ^e^	1.2 ± 0.1 ^a^	2.8 ± 0.2 ^b^	5.1 ± 5.6 ^CDE^

Different capital letters indicate significant differences among tested plants, and lowercase letters indicate significant differences among the solvent at *p* = 0.05 according to the LSD test. F—flowers; L—leaves, R—roots.

**Table 2:** The total flavonoid content in plant extracts in relation to solvent type.

Plant	Solvent										$\bar{x}$
	W	MW	MWA	MA	EW	EWA	EA	AWA2	AWA05	AWA02	
	mg QE·g^−1^ Dry Mass										
Common chamomile	10.3 ± 1.1 ^a^	11.0 ± 1.4 ^a^	16.7 ± 1.7 ^b^	22.9 ± 1.1 ^c^	21.4 ± 1.1 ^c^	27.6 ± 1.9 ^d^	22.3 ± 1.3 ^c^	33.1 ± 1.3 ^e^	45.4 ± 4.7 ^f^	34.8 ± 1.5 ^e^	24.5 ± 10.7 ^DE^
Common dandelion	4.0 ± 1.5 ^c^	3.2 ± 1.0 ^b^	1.7 ± 1.1 ^a^	5.1 ± 1.1 ^d^	2.1 ± 1.0 ^a^	5.0 ± 1.1 ^d^	3.1 ± 1.2 ^b^	6.6 ± 1.1 ^f^	5.6 ± 1.2 ^e^	2.8 ± 1.4 ^b^	3.9 ± 1.6 ^A^
Common hop	4.0 ± 1.4 ^b^	6.6 ± 1.2 ^c^	3.4 ± 1.2 ^a^	18.7 ± 1.5 ^f^	10.0 ± 1.1 ^d^	12.2 ± 1.2 ^e^	18.6 ± 1.4 ^f^	33.6 ± 1.3 ^i^	19.8 ± 1.4 ^g^	20.7 ± 1.1 ^h^	14.8 ± 9.0 ^BC^
Common nettle	5.1 ± 1.3 ^a^	5.5 ± 1.5 ^a^	3.6 ± 1.3 ^a^	5.9 ± 1.7 ^a^	4.0 ± 1.1 ^a^	4.0 ± 2.5 ^a^	12.8 ± 1.4 ^b^	12.2 ± 1.1 ^b^	20.4 ± 7.4 ^c^	26.7 ± 1.1 ^d^	10.0 ± 7.9 ^AB^
Common tansy	2.4 ± 1.8 ^a^	7.8 ± 1.1 ^b^	36.5 ± 1.5 ^g^	53.3 ± 1.5 ^i^	10.8 ± 1.3 ^c^	51.6 ± 1.8 ^h^	55.2 ± 1.6 ^j^	33.0 ± 1.5 ^f^	31.7 ± 1.3 ^e^	19.8 ± 1.2 ^d^	30.2 ± 18.8 ^EF^
Common yarrow	4.6 ± 1.1 ^a^	5.2 ± 1.3 ^ab^	9.3 ± 1.2 ^d^	16.2 ± 1.1 ^e^	5.7 ± 1.3 ^b^	7.9 ± 1.1 ^c^	9.9 ± 1.1 ^d^	26.2 ± 2.1 ^f^	59.4 ± 1.2 ^g^	63.1 ± 1.9 ^h^	20.8 ± 21.5 ^CD^
Eucalyptus nicholii	7.7 ± 2.3 ^a^	11.4 ± 1.1 ^b^	58.3 ± 1.2 ^e^	57.3 ± 2.4 ^e^	28.9 ± 1.2 ^c^	30.5 ± 1.2 ^d^	30.6 ± 1.5 ^d^	69.7 ± 1.5 ^f^	81.7 ± 1.6 ^g^	87.2 ± 1.7 ^h^	46.3 ± 27.4 ^G^
Peppermint	2.9 ± 1.0 ^a^	11.8 ± 1.1 ^b^	18.8 ± 1.3 ^d^	24.7 ± 1.1 ^f^	21.9 ± 1.1 ^e^	31.4 ± 1.1 ^h^	15.0 ± 1.3 ^c^	27.6 ± 1.1 ^g^	35.8 ± 1.2 ^i^	47.0 ± 1.1 ^j^	23.7 ± 12.2 ^DE^
Pot marigold	10.7 ± 2.4 ^a^	10.8 ± 1.7 ^a^	9.4 ± 8.7 ^a^	19.8 ± 1.2 ^bc^	16.7 ± 1.1 ^b^	16.6 ± 1.2 ^b^	22.0 ± 1.1 ^cd^	41.0 ± 2.2 ^f^	24.9 ± 1.3 ^d^	32.5 ± 1.7 ^e^	20.4 ± 10.0 ^CD^
St. John’s wort	7.0 ± 1.8 ^a^	26.4 ± 1.1 ^c^	15.2 ± 1.2 ^b^	28.6 ± 1.5 ^d^	26.5 ± 1.0 ^c^	37.7 ± 1.3 ^f^	29.6 ± 1.4 ^e^	66.0 ± 1.8 ^i^	60.3 ± 1.2 ^h^	58.5 ± 1.4 ^g^	35.6 ± 19.2 ^F^
Valerian	0.9 ± 0.1 ^a^	1.3 ± 0.1 ^a^	4.0 ± 0.1 ^b^	5.1 ± 0.0 ^c^	6.2 ± 1.2 ^d^	8.0 ± 1.1 ^f^	6.5 ± 1.1 ^de^	9.2 ± 1.5 ^g^	7.6 ± 1.5 ^f^	6.9 ± 1.3 ^e^	5.6 ± 2.7 ^A^
Wormwood	3.4 ± 1.1 ^a^	4.2 ± 1.1 ^b^	5.2 ± 1.1 ^c^	5.5 ± 1.1 ^d^	6.2 ± 1.1 ^e^	11.4 ± 1.1 ^h^	7.6 ± 1.3 ^f^	12.4 ± 1.1 ^i^	11.2 ± 1.1 ^h^	8.2 ± 1.2 ^g^	7.5 ± 3.1 ^AB^

Different letters indicate significant differences at *p* = 0.05 according to the LSD test.

**Table 3:** The total polyphenol content in plant extracts in relation to solvent type.

Plant	Solvent										$\bar{x}$
	W	MW	MWA	MA	EW	EWA	EA	AWA2	AWA05	AWA02	
	mg GAE·g^−1^ Dry Mass										
Common chamomile	54.8 ± 1.7 ^a^	105.2 ± 1.5 ^c^	124.3 ± 1.5 ^e^	120.0 ± 2.3 ^d^	131.4 ± 1.7 ^g^	126.6 ± 1.1 ^f^	74.6 ± 1.8 ^b^	148.1 ± 3.2 ^i^	143.7 ± 1.4 ^h^	150.4 ± 1.5 ^j^	117.9 ± 30.4 ^D^
Common dandelion	9.0 ± 1.1 ^a^	27.9 ± 1.1 ^e^	30.1 ± 1.1 ^f^	30.0 ± 1.1 ^f^	22.6 ± 1.2 ^b^	26.3 ± 1.2 ^d^	23.7 ± 1.3 ^c^	37.9 ± 1.4 ^g^	50.2 ± 1.3 ^h^	29.7 ± 1.3 ^f^	28.7 ± 10.2 ^A^
Common hop	46.4 ± 1.0 ^a^	98.5 ± 2.4 ^d^	71.3 ± 1.8 ^b^	115.8 ± 1.2 ^f^	106.0 ± 1.6 ^e^	74.3 ± 1.7 ^c^	136.6 ± 1.2 ^h^	117.4 ± 2.5 ^f^	131.6 ± 3.2 ^g^	158.6 ± 4.3 ^i^	105.6 ± 32.8 ^CD^
Common nettle	26.4 ± 6.1 ^bc^	29.3 ± 2.0 ^c^	23.7 ± 1.2 ^ab^	56.7 ± 1.4 ^g^	22.3 ± 2.2 ^a^	26.6 ± 1.3 ^bc^	41.3 ± 1.9 ^e^	30.3 ± 1.4 ^cd^	47.0 ± 5.9 ^f^	34.9 ± 1.6 ^d^	42.7 ± 11.0 ^AB^
Common tansy	74.3 ± 1.4 ^a^	97.1 ± 1.2 ^b^	119.8 ± 1.7 ^c^	270.1 ± 4.1 ^i^	156.1 ± 2.6 ^d^	224.1 ± 2.2 ^e^	286.9 ± 3.2 ^j^	264.2 ± 2.1 ^h^	253.8 ± 3.6 ^g^	242.6 ± 3.4 ^f^	198.9 ± 76.6 ^F^
Common yarrow	42.1 ± 1.2 ^b^	22.6 ± 2.0 ^a^	56.8 ± 1.2 ^c^	58.3 ± 2.5 ^c^	22.6 ± 1.3 ^a^	57.0 ± 1.2 ^c^	83.3 ± 2.4 ^d^	193.0 ± 3.1 ^e^	231.5 ± 2.5 ^g^	216.0 ± 1.7 ^f^	89.5 ± 79.1 ^C^
Eucalyptus nicholii	211.2 ± 1.4 ^a^	278.8 ± 8.1 ^c^	337.1 ± 5.4 ^d^	380.6 ± 7.6 ^e^	240.6 ± 2.1 ^b^	292.3 ± 1.7 ^c^	321.3 ± 2.5 ^d^	426.2 ± 6.1 ^f^	444.6 ± 17.2 ^g^	462.6 ± 25.9 ^h^	339.5 ± 84.2 ^G^
Peppermint	35.0 ± 1.1 ^a^	64.5 ± 1.4 ^b^	108.4 ± 1.1 ^e^	193.7 ± 1.7 ^j^	92.7 ± 1.9 ^d^	185.0 ± 1.9 ^i^	87.1 ± 1.2 ^c^	116.6 ± 1.4 ^f^	118.0 ± 1.4 ^g^	126.6 ± 1.7 ^h^	112.8 ± 47.1 ^CD^
Pot marigold	35.9 ± 1.7 ^a^	83.4 ± 1.2 ^c^	89.9 ± 2.0 ^d^	97.8 ± 1.7 ^e^	83.6 ± 3.8 ^c^	59.2 ± 1.2 ^b^	90.9 ± 1.3 ^d^	188.5 ± 2.8 ^h^	165.1 ± 2.7 ^f^	170.6 ± 2.7 ^g^	106.4 ± 48.9 ^CD^
St. John’s wort	80.0 ± 1.0 ^a^	117.2 ± 1.5 ^c^	114.0 ± 58.3 ^bc^	153.7 ± 1.5 ^d^	84.1 ± 1.3 ^ab^	210.8 ± 1.5 ^e^	178.2 ± 2.3 ^d^	239.1 ± 2.0 ^e^	217.7 ± 2.4 ^e^	273.5 ± 2.3 ^f^	166.8 ± 67.0 ^E^
Valerian	16.5 ± 1.1 ^a^	38.9 ± 2.0 ^b^	45.6 ± 1.2 ^bc^	18.8 ± 1.1 ^a^	43.4 ± 1.2 ^bc^	54.6 ± 2.1 ^bc^	55.7 ± 7.7 ^c^	88.7 ± 2.7 ^d^	82.7 ± 3.6 ^d^	73.8 ± 28.6 ^d^	51.9 ± 25.0 ^AB^
Wormwood	27.6 ± 3.6 ^a^	49.7 ± 1.1 ^b^	54.2 ± 1.4 ^c^	66.7 ± 1.2 ^e^	27.2 ± 1.2 ^a^	63.3 ± 2.8 ^d^	68.2 ± 1.5 ^e^	53.5 ± 1.4 ^c^	89.1 ± 1.8 ^g^	79.9 ± 2.7 ^f^	57.9 ± 19.4 ^B^

Different letters indicate significant differences at *p* = 0.05 according to the LSD test.

**Table 4:** Characterization of compounds isolated from the plant acetone extracts by the HPTLC analyses.

Rf	White Light	UV 254 nm	UV 366 nm Before Derivatization	UV 366 nm After Derivatization	Component Identification	Common Chamomile	Common Dandelion	Common Hop	Common Nettle	Common Tansy	Common Yarrow	Eucalyptus Nicholii	Peppermint	Pot Marigold	St. John’s Wort	Valerian	Wormwood
0.030	-	q	blue	-	phenolic acid					+					+	+	
0.040	yellow	q	blue	yellow	flavonoid					+	+				+	+	
0.042	gray	q	-	-	unidentified							+					
0.067	yellow	q	-	orange	flavonoid						+						
0.082	-	q	blue	light blue	phenolic acid						+			+			+
0.097	yellow	q	yellow-blue	yellow						+							
0.100	-	q	navy blue	navy blue	phenolic acid	+			+								
0.109	yellow	q	-	orange	flavonoid									+			
0.124	yellow	q	-	orange							+						
0.140	yellow	q	-	yellow	flavonoid									+			
0.167	-	q	blue	light blue	phenolic acid				+	+							
0.197	-	q	navy blue	navy blue	phenolic acid				+								
0.206	-	q	-	light blue	phenolic acid												+
0.225	-	q	blue	light blue						+	+						
0.243	yellow	q	blue	orange	flavonoid										+		
0.273	yellow	q	-	orange	rutin *			+	+	+			+	+	+		
0.279	-	q	navy blue	light blue			+										
0.279	-	q	navy blue	navy blue	phenolic acid							+					
0.297	-	q	blue	blue	phenolic acid				+								
0.319	-	q	blue	blue	phenolic acid		+		+								
0.322	yellow	q	-	-	flavonoid	+											
0.334	pink	q			unidentified								+				
0.343	-	q	blue	light blue	chlorogenic acid *	+				+	+	+		+		+	+
0.358	yellow	q	blue	orange	flavonoid					+	+	+					
0.367	-	q	navy blue	navy blue	phenolic acid				+					+			
0.376	-	q	blue	light blue	phenolic acid	+		+		+					+		
0.398	yellow	q	-	orange	flavonoid					+	+				+		
0.404	-	q	light blue	light blue													+
0.449	yellow	q	-	orange	hyperoside *			+				+			+		
0.479	yellow	q	-	orange	flavonoid	+		+		+			+		+		
0.521	-	q	navy blue	light blue	cynarine *					+						+	
0.549	yellow	q	navy blue	orange	flavonoid	+		+				+					
0.589	yellow	q	-	yellow	kaempferol-3-glucoside *					+							
0.592	-	q	navy blue	light blue	phenolic acid	+										+	
0.604	-	q	navy blue	light blue	phenolic acid	+											
0.640	-	q	blue	light blue	phenolic acid								+				
0.671	-	q	navy blue	light blue	phenolic acid		+										
0.710	gray	q	red	red	chlorophyll										+		
0.713	-	q	blue	light blue	phenolic acid				+								
0.756	-	q	blue	light blue		+	+			+	+					+	+
0.807	-	q	blue	light blue	phenolic acid								+				
0.807	-	q	blue	navy blue	phenolic acid							+					
0.828	-	q	navy blue	navy blue	phenolic acid				+					+			
0.868	-	q	blue	light blue	phenolic acid	+					+		+	+	+	+	+
0.875	yellow	q		orange	luteolin *					+							
0.895	yellow	q	light blue	orange	quercetin *							+			+		
0.922	gray-green	q	red	red	chlorophyll	+		+	+	+	+	+	+		+		+
0.980	yellow	q	blue	light blue	flavonoid			+									
1.000	-	q	red	red	chlorophyll	+		+									+

Rf—retardation factor, q—fluorescence quenching, +—band present in sample, -—band absent in sample, *—identification based on comparison with analytical standard.

**Table 5:** List of plants and their parts used against *A. tumefaciens*.

Common Name	Scientific Name	Family	Parts Used
Common chamomile	*Matricaria chamomilla* L.	*Asteraceae*	Flowers
Common dandelion	*Taraxacum officinale* F.H. Wiggers coll.	*Asteraceae*	Roots
Common hop	*Humulus lupulus* L.	*Cannabaceae*	Flowers
Common nettle	*Urtica dioica* L.	*Urticaceae*	Leaves
Common tansy	*Tanacetum vulgare* L.	*Asteraceae*	Leaves
Common yarrow	*Achillea millefolium* L.	*Asteraceae*	Leaves
Eucalyptus nicholii	*Eucalyptus nicholii* L.	*Myrtaceae*	Leaves
Peppermint	*Mentha piperita* L.	*Lamiaceae*	Leaves
Pot marigold	*Calendula officinalis* L.	*Asteraceae*	Flowers
St. John’s wort	*Hypericum perforatum* L.	*Hypericaceae*	Leaves
Valerian	*Valeriana officinalis* L.	*Valerianaceae*	Roots
Wormwood	*Artemisia absinthium* L.	*Asteraceae*	Leaves

**Table 6:** Solvents used for active compound extraction from plant material.

Solvent Symbol	Solvent Composition
W	Water (100.0%)
MW	Methanol/water (50.0/50.0%)
MWA	Methanol/water/acetic acid (50.0/49.5/0.5%)
MA	Methanol/acetic acid (99.5/0.5%)
EW	Ethanol/water (50.0/50.0%)
EWA	Ethanol/water/acetic acid (50.0/49.5/0.5%)
EA	Ethanol/acetic acid (99.5/0.5%)
AWA2	Acetone/water/acetic acid (70.0/28.0/2.0%)
AWA05	Acetone/water/acetic acid (70.0/29.5/0.5%)
AWA02	Acetone/water/acetic acid (70.0/29.8/0.2%)

## Data Availability

The original contributions presented in this study are included in the article. Further inquiries can be directed to the corresponding author.
